# Density functional theory and machine learning for electrochemical square-scheme prediction: an application to quinone-type molecules relevant to redox flow batteries†

**DOI:** 10.1039/d3dd00091e

**Published:** 2023-09-12

**Authors:** Arsalan Hashemi, Reza Khakpour, Amir Mahdian, Michael Busch, Pekka Peljo, Kari Laasonen

**Affiliations:** a Department of Chemistry and Material Science, School of Chemical Engineering, Aalto University 02150 Espoo Finland arsalan.hashemi@aalto.fi; b Institute of Theoretical Chemistry, Ulm University Albert-Einstein Allee 11 89069 Ulm Germany; c Research Group of Battery Materials and Technologies, Department of Mechanical and Materials Engineering, Faculty of Technology, University of Turku 20014 Turun Yliopisto Finland

## Abstract

Proton–electron transfer (PET) reactions are rather common in chemistry and crucial in energy storage applications. How electrons and protons are involved or which mechanism dominates is strongly molecule and pH dependent. Quantum chemical methods can be used to assess redox potential (*E*_red._) and acidity constant (p*K*_a_) values but the computations are rather time consuming. In this work, supervised machine learning (ML) models are used to predict PET reactions and analyze molecular space. The data for ML have been created by density functional theory (DFT) calculations. Random forest regression models are trained and tested on a dataset that we created. The dataset contains more than 8200 quinone-type organic molecules that each underwent two proton and two electron transfer reactions. Both structural and chemical descriptors are used. The HOMO of the reactant and LUMO of the product participating in the oxidation reaction appeared to be strongly associated with *E*_red._. Trained models using a SMILES-based structural descriptor can efficiently predict the p*K*_a_ and *E*_red._ with a mean absolute error of less than 1 and 66 mV, respectively. Good prediction accuracy of *R*^2^ > 0.76 and >0.90 was also obtained on the external test set for *E*_red._ and p*K*_a_, respectively. This hybrid DFT-ML study can be applied to speed up the screening of quinone-type molecules for energy storage and other applications.

## Abstract 2

Computational high-throughput is used to evaluate proton–electron transfer reactions of quinone-type compounds that are potentially useful for energy storage.

## Graphical abstract

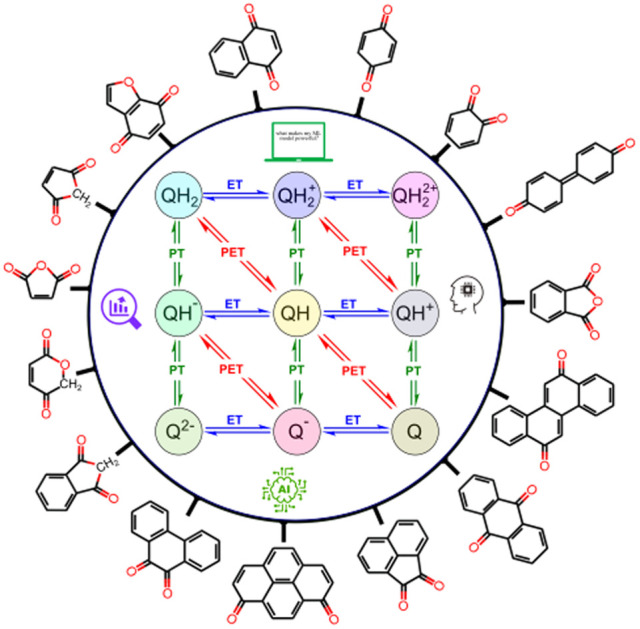

## Introduction

Proton–electron transfer (PET) is a fundamental reaction in electrochemistry,^1–3^ biochemistry,^4,5^ material science,^6,7^ and in some other fields.^8,9^ To give one example, we can consider the charging and discharging processes in aqueous redox flow batteries,^10–14^ in which the two elementary steps, *i*.*e*., electron transfer (ET) and proton transfer (PT), are taken either competitively or jointly to interconvert electricity and chemical energy. Therefore, understanding the pathways from reactants to products through PET reactions is crucial to understand flow battery performance.

In the recent development of redox flow batteries (RFB), water-soluble organic molecules are at the forefront of attention due to their affordability, safety, and structural diversity.^15,16^ In water, pH will impact the protonated/deprotonated form of the reduced or oxidized molecules participating in the redox reaction.^17–20^ When the proton concentration is lowered, *i*.*e*., the pH is raised, even strong bases may not be protonated. Hence, pH-potential diagrams of the electrolyte species, known as Pourbaix diagrams,^21^ should be assessed early in the battery design process. The Pourbaix diagram can be constructed using the redox potential (*E*_red._) and p*K*_a_ of the involved species.

Rational designing and testing of materials in order to find better candidates is the focus of molecular engineering. Organic molecules can be modified either by their functional groups or their backbones. Numerous experimental studies have been performed to improve solubility, electrochemical redox properties, and cycling stability by modifying functional groups.^19,20,22–26^ For instance, Wedege *et al.*^20^ and Wiberg *et al.*^22^ in separate studies observed that solubility and redox potential is influenced by the position, type, and number of functional groups. Moreover, another study showed that heterocycles, where one carbon is substituted by oxygen, sulfur, and nitrogen, can change charge states and improve reactivity.^27^ Nevertheless, the experimental verification of the RFB active molecules is slow, especially if new molecules need to be synthesised, the computational pre-screening is very useful.

In the past few years, there have been several computational high-throughput studies that combined quantum mechanical density functional theory (DFT) and machine learning (ML) to find potential candidates for flow batteries.^28–43^ Mostly, for sets of limited isomeric backbones, these studies primarily focused on solubility and redox potential of the hydrogen atom transfer reaction at pH = 0 when functional groups were decorated. ChemAxon software^44^ has also developed powerful models for predicting p*K*_a_ and solubility. However, its accuracy always depends on both the quality and the size of the dataset. To the best of our knowledge, the PET reaction mechanism has not yet been studied in a systematic high-throughput manner.

This contribution attempts to carefully map the chemical and structural characteristics of a library of quinone-type compounds^45,46^ to their square-scheme representations of the potential for ET and PT. In this report, we provide (i) a deeper understanding of molecule design for energy storage applications from a screening of large chemical space and (ii) a trained ML model to predict so far unknown redox-active molecules.

## Theoretical framework

**Fig. 1:**
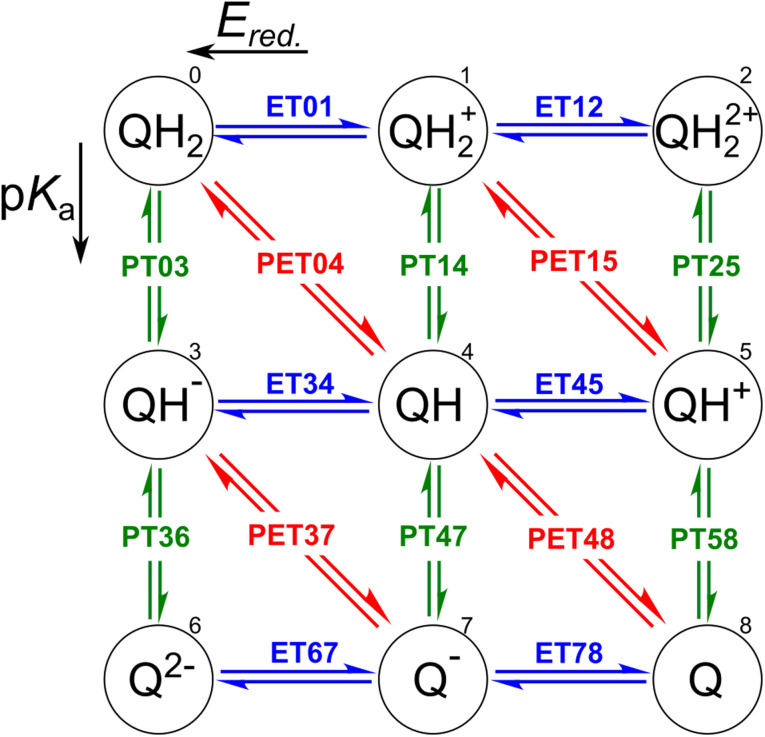
Square representation for two-proton two-electron transfer redox reactions. Electron transfer (ET) and proton transfer (PT) reactions are represented by blue horizontal and green vertical arrows, respectively. The diagonal red arrows indicate proton–electron transfer (PET) reactions. Each state was numbered to simplify demonstrations of *E*_red._ and p*K*_a_ involved in the reactions.

The redox properties of a molecule can be described in two ways: as a proton–electron transfer (PET; red arrows in Fig. 1), or as a decoupled sequence of electron transfer (ET; blue arrows in Fig. 1) and proton transfer (PT; green arrows in Fig. 1). In this study, a molecule QH_2_ (Q) is oxidized (reduced) to Q (QH_2_) by releasing (accepting) two electrons and two protons as follows:

1 QH_2_ ⇌ Q + 2H^+^ + 2e^−^,

which results in multiple coupled or decoupled pathways, as shown in Fig. 1. In practice, the required proton (H^+^) is transferred from an aqueous solution, while electron (e^−^) is obtained from an electrode.

In order to assess the redox reaction (eqn (1)), it is necessary to calculate Gibbs free energy change Δ*G* at each ET/PT step: Δ*G* leads to the measurable reduction potential *E*_red._ and acidity constant p*K*_a_ for the ET and PT reactions, respectively. One can also predict the number of transferred electrons and protons at the given solution pH and electrode potential using these quantities.

Clearly, the most delicate part of Δ*G* calculation is to determine the energetics of the involved proton and electron transfers without explicit solvent and physical electrode in our model system. We use a three-step protocol to determine Δ*G* of PET, PT, and ET reactions in order:

(i) Following earlier works, the energetics of the PET step can be computed using the computational standard hydrogen electrode (SHE).^47,48^ According to this method, the energetic of a PET step, *e*.*g*. for QH → Q + H^+^ + e^−^, under standard condition, *i*.*e*. pH = 0, is given by2
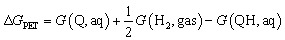
where, *G*(QH, aq) and *G*(Q, aq) correspond to the Gibbs free energies of the reduced species (QH) and the oxidized species (Q), respectively, in the aqueous phase. Hydrogen dimer in the gas phase is considered as a reference for electron and proton energies, *i*.*e*. *G*(H_2_, gas)/2.

(ii) The Δ*G* of a PT step, *e*.*g*. for QH^+^ → Q + H^+^, is computed using the isodesmic method, as follows:

3 Δ*G*_PT_ = *G*(Q, aq) + *G*(H^+^, aq) − *G*(QH^+^, aq).

It leads us to calculate the measurable p*K*_a_ value as follows:4
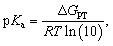
where *R* and *T* are the general gas constant and temperature, respectively. While the Gibbs free energies of the deprotonated (Q) and protonated (QH^+^) species are easily examined in eqn (3), the *G*(H^+^) is a challenging part to assess. To address this issue, we use the experimental p*K*_a_ as a reference.^49,50^ Herein, formic acid (HCOOH) dissociation reaction with p*K*^ref.^_a_ = 3.77 is employed:^51^

5 HCOOH ⇒ HCOO^−^ + H^+^.

Using eqn (4) and, subsequently, eqn (3), the values of Δ*G*_PT_ and *G*_H^+^_ are calculated. Having the latter quantity enables us to compute the p*K*_a_ value of any PT reaction.

The obtained p*K*_a_ values then need to be scaled to overcome shortcomings of the implicit solvation model^52^ used in the present study^53–56^ as follows:

6 p*K*^scaled^_a_ = 0.49p*K*_a_ + 3.2,

where p*K*^scaled^_a_ is correlated to the experimental measurements. We get Δ*G*^scaled^_PT_ when eqn (6) is put into eqn (4).

(iii) The potential associated with the ET step is finally computed as the difference between the energetics of the PET and the PT step as^57,58^

7 Δ*G*_ET_ = Δ*G*_PET_ − Δ*G*^scaled^_PT_.

In addition to Δ*G*_PET_, Δ*G*_ET_ values are also referenced to the computational standard hydrogen electrode (SHE).

Generally, the accuracy of the calculated data in comparison to experiments depends essentially on the adopted approximations and the electronic structure calculations to compute Gibbs free energy values. In other words, a negligible systematic error can be expected at this level of calculations.

It is worth noting that, in accordance with the IUPAC convention,^59^ we state every electrochemical potential as a reduction reaction potential. Hereafter, *E*^0^ = Δ*G*_PET_/*e* and *E*_red._ = Δ*G*_ET_/*e* are indicators for H atom and e^−^ affinity of molecule Q participating oxidation reaction (*i*.*e*. eqn (1)), respectively. The theoretical framework employed in this study has previously been established and extensively used for the p*K*_a_, *E*_red._, and *E*^0^ estimations.^43,53,60–62^ When compared to the experiments, the predictability of the DFT-calculated *E*_red._ and *E*^0^ is very good.^43^

## CompBatPET database

Our CompBatPET database consists of 8213 organic compounds undergoing two-proton two-electron reactions. The data are stored in the comma-separated values (CSV) file format and XYZ file. One data set was made for each ET, PT, and PET reaction, containing the molecular weights, cavity volumes, highest occupied orbital energies (HOMOs), lowest unoccupied orbital energies (LUMOs), and simplified molecular input line entry system (SMILES) string representations of only the reactants involved in the oxidation and deprotonation reactions. Open Babel^63^ software was used to record the SMILES. Since SMILES contains information about H-bond changes but not net electron charges, we also add the sample's net charge to the data sets. Note that the molecular weights, cavity volumes, and molecular orbital energies are reported in atomic units, Å^3^, and Hartree, respectively.

Each reactant of the oxidation reaction, namely QH_2_, QH_2_^+^, QH^−^, QH, Q^2−^, and Q^−^, is accompanied by its target variable *E*_red._. While, p*K*_a_ is featured by the protonated samples found in the QH_2_, QH_2_^+^, QH_2_^2+^, QH^−^, QH, and QH^+^ forms. Deprotonation converts alcohol (C–OH) fragments into ketone (C=O). In addition, QH_2_, QH_2_^+^, QH^−^, and QH are considered as reactants for PET reactions when *E*^0^ is the target variable. All the numerical values are imported up to three digits after the decimal point.

**Fig. 2:**
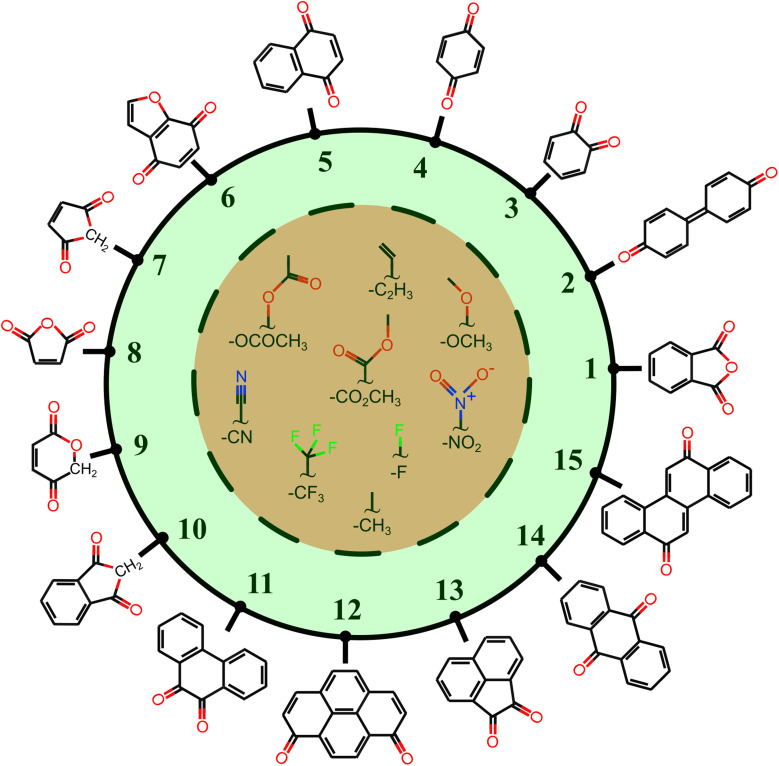
The core structure of studied compounds accompanied by 9 organic functional groups. The cores are numbered from 1 to 15. The functional groups are demonstrated in the central part of the picture. The molecular space is constructed by the combinatorial attachment of one or two functional groups.

All the molecules in the database are built upon a group of 15 core structures, as schematized in Fig. 2, decorated by –CH_3_, –CF_3_, –OCH_3_, –C_2_H_3_, –F, –CN, –NO_2_, –OCOCH_3_, and –CO_2_CH_3_ functional groups. These functional groups do not participate in the PT reaction, but they have either electron-donating or electron-withdrawing characteristics. The IUPAC names of the core structures can be found in Fig. S1 of ESI.†

Each core is manually designed by one or two functional groups. The task of functional group enumeration is performed using the Maestro modeling interface of Schrödinger Material Science Suite (SMSS).^64^ The combinatorial search of molecular structures results in 8213 molecules with a diversity of (1:254, 2:605, 3:267, 4:139, 5:644, 6:523, 7:55, 8:55, 9:100, 10:271, 11:1187, 12:970, 13:649, 14:604, and 15:1890). We provide open access to full source code and data sets at https://doi.org/10.5281/zenodo.7952777.

## Computational details

All DFT calculations were performed using Gaussian 16 revision C.01 (ref. 65) software. First, the semi-empirical PM7 (ref. 66) method was used to optimize the geometry of molecular structures solvated in PCM^67,68^ implicit water model. Note that for each molecule the initial atomic coordinates were prepared by Maestro, as mentioned before, and visually inspected by the authors. At this stage, the energetics of two different structures were compared in order to determine the most stable tautomers of the individual QH^−^, QH, and QH^+^ forms. After the structure optimization, harmonic vibrational frequencies were assessed for free energy stability evaluation. The structure of a molecule with an imaginary frequency less than −200 cm^−1^ was modulated and reoptimized until the stable structure was obtained. Then, the total energy was computed for each optimized structure using M06-2X^69^ together with a Def2-TZVP basis set^70^ and the more accurate SMD solvation model. This exchange–correlation functional was found to provide the best accuracy for predicting redox potentials of organic molecules.^60^ To compute the Gibbs free energy, thermal correction to Gibbs free energy and total energy were obtained from the computationally cheaper PM7 (the first step) and high accuracy M06-2X calculations (the second step), respectively. The thermal correction includes the zero-point vibrational energy as well as vibrational enthalpy and entropic contribution to the free energy. This procedure leads to moderate computational cost and has been validated previously.^36^

For high-throughput screening, we used Random Forest Regressor (RFR) as implemented in Scikit-learn package^71^ of Python. The optimal set of hyperparameters was computed using a cross-validation score over a grid of predefined space. More specifically, the training data was split into ten groups, or folds, where nine were used to train the model and one is used to evaluate its performance. Mean squared error (MSE) was used as an evaluation metric for hyperparameter optimization. To train our RFR model, the data set was initially randomized, then 80% of the data were used for training the model, while 20% for validation. Subsequently, the trained models were used to predict the electrochemical square-schemes, *i.e. E*_red._, p*K*_a_, and *E*^0^ values. Note, for each target variable listed above a separate model is trained.

The isomeric SMILES representation of the molecules is transformed into a bit-vector using extended-connectivity fingerprints (ECFPs) algorithm.^72,73^ We used RDKit package^74^ to rapidly calculate ECFPs. In general, they work by circularly analyzing the environment of each atom and, then, hashing the information to create the fingerprints (FPs). A radius of 3 nearest neighbors was used. Chirality was also considered for the FP assessment. It is critical for backbone 2. The bit vector contains 0 and 1 with a length of 1024 for each molecule.

It is often difficult to interpret the output of ML models because the methods provide very little information on the descriptors' contribution to the output. We employed Shapley additive explanation^75^ (SHAP) to test whether our chemical intuition agrees with the results of the ML model. Feature importance analysis is also performed. Additionally, Matplotlib,^76^ Seaborn,^77^ and Pandas^78^ as the main Python visualization and data manipulation libraries were used. The ESI† provides more details on the package dependencies as well as a suitable environment for ML calculations.

## Results and discussion

### Data analysis

**Fig. 3:**
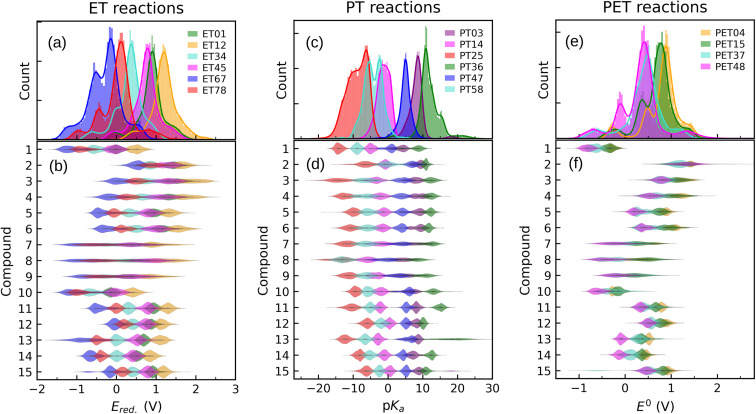
Data distribution accompanied by violin plots showing the contributions of each compound (*i* = 1, …, 15) for ((a) and (b)) ET, ((c) and (d)) PT, and ((e) and (f)) PET. The reactions in the plots are shown in Fig. 1. The ease of ET, PT, and PET reactions is indicated by the target values *E*_red._, p*K*_a_, and *E*^0^, respectively. *E*_red._ and *E*^0^ were referenced to the SHE.

In order to inspect the data, we examine the distribution of *E*_red._, p*K*_a_, and *E*^0^ as shown in Fig. 3. Detailed statistical data was presented in Table S1.† In chemistry, *E*_red._ is defined as the tendency of a molecule to accept an electron: a more positive *E*_red._ indicates a stronger electron-accepting ability of the reactant participating in the reduction reactions. The distribution of *E*_red._ potential is shown in Fig. 3(a): (i) as a result of different ET reactions, there are six distinct peaks, (ii) each ET case shows a multimodal distribution with a dominant peak in the center, (iii) when the number of protons is constant, the second electron donation reaction occurs at a lower *E*_red._ value, (iv) protonation makes reduction reaction thermodynamically more favorable, and (v) *E*_red._ ranges from −1.629 to 2.426 V_SHE_.

The impact of the core architecture and functionalization on the *E*_red._ is demonstrated in Fig. 3(b). A violin plot depicts the shape of the data distribution: the broader section of a violin represents a higher variation of *E*_red._. A narrow bit thick in the middle of the plot indicates that data is highly concentrated around the median. One can also say that a fairly uniform violin plot with long tails indicates (cores 7 to 9) that functional type and/or position have a significant impact on that type of core. When compared to other counterparts, cores 1 and 10 have the highest electron-donating characteristic. ET reduction reactions are thermodynamically less favorable for molecules in these two families. We found that small single-ring compounds 3, 4, 7, 8, and 9 are sensitive to functional group addition. This is not very surprising since inductive/mesomeric effects will pump in or remove the electron density for carbons.^79^

Fig. 3(c) depicts the p*K*_a_ distribution: each of the PT03, PT14, and PT47 deprotonation reactions is determined by a unimodal peak, while the others are bimodal. The conversion to a ketone through oxidation increases the acidity of the molecules while deprotonation decreases it. This is in line with common trends in acid–base chemistry which clearly indicate that ketones are very strong acids.^79^ Additional negatively charged or the removal of positively charged functional groups reduce the acid strength (*i*.*e*. increase the p*K*_a_).^61^ Therefore, compounds in QH_2_^2+^ and QH^−^ states represent the strongest and weakest acids, respectively. Those with negative p*K*_a_ are unstable in normal solutions and they release proton into the solution even at pH = 0. As a general rule, deprotonation occurs whenever the pH of the solution is greater than the p*K*_a_ of the solvated molecule. Note that not all the studied QH_2_ terminate to Q at pH = 0. It is seen from the partially positive p*K*_a_ of QH^+^ (*i.e.* PT58 reaction). In other words, some molecules are deprotonated in a less acidic media.

For each backbone, the p*K*_a_ values are shown in Fig. 3(d). The acidity strength of the reactants participating in reactions PT25, PT58, PT14, PT47, PT03, and PT36 decreases systematically, with the exception of backbones 2 and 8 where PT58 data overlaps with PT14 and PT25, respectively. The p*K*_a_ of compound 13 involved in reaction PT36 is widely distributed. With all data taken into account, p*K*_a_ ranges from −22 to 30.

The data distribution relative to the PET reactions is multimodal, as shown in Fig. 3(e). Each reaction showed a dominant peak centered at a positive value. There is a resemblance between the energy spectrum of PET15 and PET37 with that of PET04, respectively, and PET48. Similarities in the proton states of the reactants may explain it. *E*^0^ ranges from −1.222 to 2.722 V_SHE_.

By removing hydrogen atoms, molecules in the form of QH_2_ are converted into partially and fully oxidized forms of QH and Q, respectively. Another possibility is that QH_2_ initially undergoes an ET or PT reaction, then a PET, such as PET15 and PET37. Whenever these reactions take place, the redox potential in water at pH = 0 equals *E*^0^. According to our results, the PET reactions mostly take place within the water electrochemical potential window of *ca.* −1.5 to +1.5 V_SHE_.^80^Fig. 3(f) illustrates the backbone effects and demonstrates that compounds 1 and 10 are highly driven to lose hydrogen atoms. Compounds 7, 8, and 9 partially and to a lesser extent exhibit the same behavior. Otherwise, the PET reactions are endothermic. We see no outliers in the data, and it is well-prepared for further analysis.

### Machine learning

Property- and structure-based descriptors are two alternative features that are used to train ML models. DFT calculations are used to determine chemical characteristics such as molecular orbitals. Whereas, the structure-based descriptor is produced directly from SMILES, which includes information about the topology, connectivity, and subfragment of the molecules. The latter enables high-throughput screening of candidates at a significantly lower computational cost, *i.e.* without DFT calculations.

The RFR models are trained with 200 trees. Cross-validation shows that the default values can be used for the remaining hyperparameters. Model performance was evaluated using a coefficient-of-determination (*R*^2^), root-mean-squared-error (RMSE), and mean-absolute-error (MAE). These performance metrics were defined in the ESI.† The performance assessment is performed five times: every time the data set is split, the model is trained and tested, and the worst performance result is reported.

For *E*_red._ and p*K*_a_, the data set of chemical properties contains 49 278 samples, while the structure-based one contains 49 271 samples altogether. There are seven fewer samples in the second data set because of the molecules' rotational symmetry. The SMILES duplicate check found them, and they were removed. Additionally, the relevant information of QH_2_, QH_2_^+^, QH^−^, and QH compounds was stored for predicting *E*^0^. There are 32 865 samples in this dataset.

### Property-based descriptor

In addition to HOMO, HOMO−1, LUMO, and LUMO+1 energies for spin-up and spin-down channels, the chemical parameters include net charge, number of atoms, chemical weight, and volume. Here the volume means the cavity volume used in the solvation model for each molecule. This group is called Descriptor I. All numbers were collected from SMD/M06-2X calculations.

It is clear that net charge plays a significant role in the feature space. It affects electron density, resulting in a change in the orbital energies. In order to gain insight into orbital impacts on the target values, we considered only orbital energies (*e.g.*, HOMO, HOMO−1, LUMO, LUMO+1) as Descriptor II. We also made Descriptor III which includes only HOMO. This was used for *E*_red._ and *E*^0^ predictions.

To analyze the results of ML models trained on different descriptors, the performance metrics were obtained in Table 1. The closer *R*^2^ to 1 or the lower the RMSE and MAE values are, the more accurate the model is. When considering the prediction accuracy of various models trained on the data in Descriptor I, *E*_red._, p*K*_a_, and *E*^0^ are predicted by MAE ≤ 0.062 V, ≤0.759 p*K*_a_ unit, and ≤0.106 V, respectively, which are extremely good. For the ET steps an error of roughly 0.1 V is obtained with CCSD(T) while the error bars for p*K*_a_ values are also of the same order of magnitude.^56,60^ A well-trained model and excellent correlation between features and target parameters are also shown by the RMSE and *R*^2^ values. Moving to Descriptor II feature space causes the performance of the models to somewhat deteriorate but is still comparable to chemical accuracy, *e*.*g*. the MAEs are of the order of 0.05 V. It means that a limited number of molecular orbitals carry sufficient information for ML model training.

**Table 1:** Performance of RFR models trained on the property-based feature space: test set RMSE, MAE, and *R*_tst_^2^, accompanied by train set *R*_trn_^2^ and out-of-bag (oob) *R*_oob_^2^ score. Depending on the target variable or descriptor, models 1 to 14 differ

Model	Descriptor	Target	RMSE	MAE	*R*_trn_^2^	*R*_tst_^2^	*R*_oob_^2^
1	I	*E*_red._	0.093	0.062	1.00	0.98	0.98
2	II	*E*_red._	0.102	0.068	0.99	0.97	0.97
3	III	*E*_red._	0.229	0.177	0.92	0.87	0.87
4	I	p*K*_a_	1.203	0.759	0.99	0.97	0.97
5	II	p*K*_a_	1.477	0.929	0.99	0.96	0.96
6	I	*E* ^0^	0.106	0.073	0.99	0.94	0.94
7	II	*E* ^0^	0.127	0.084	0.98	0.92	0.92
8	III	*E* ^0^	0.397	0.305	0.53	0.22	0.22
9	IV	*E*_red._	0.132	0.081	0.99	0.96	0.96
10	IV	p*K*_a_	1.406	0.909	0.99	0.97	0.97
11	IV	*E* ^0^	0.157	0.106	0.94	0.88	0.90
12	FP	*E*_red._	0.099	0.066	0.99	0.99	0.96
13	FP	p*K*_a_	1.003	0.628	0.99	0.98	0.97
14	FP	*E* ^0^	0.102	0.065	0.96	0.95	0.96

Note that by comparing the energy of the orbitals of the spin-up and spin-down channels, as shown in Fig. S2(a) and (b),† it can be seen that the HOMO spin-up channel has a higher energy level than the spin-down channel for the reactants participated in the ET and PET reactions through the oxidation reactions. Therefore, when we discuss HOMO, we are referring to the spin-up channel orbitals. Through SHAP, we carried out feature importance analysis that determines the attribution of feature variables of each sample on the model prediction.^81^ HOMO state is the most important feature in model training to predict *E*_red._ and *E*^0^, as shown in Fig. S3.† The absence of this feature in some samples can deteriorate *E*_red._ prediction beyond ±1.5 V. This value for *E*^0^ is around ±1 V. For the p*K*_a_ prediction, those orbitals positioned at the edge of the HOMO–LUMO gap are the most important.

The HOMO alone, Descriptor III, can predict *E*_red._ reasonably good even with RMSE = 0.229 and MAE = 0.177 V. Whereas, the *E*^0^ forecasting is unsatisfactory and the contribution of the other states is necessary. Plotting target value *versus* HOMO reveals more sparsity for *E*^0^ values in comparison to *E*_red._ (see Fig. S4(a) and (b)†).

We carried out comparative analyses to determine the influence of product attributes on the target values. Descriptor IV is introduced as an equivalent to Descriptor II, but it contains the product species information. In general, models 9–11 have predictability comparable to those trained on reactants' feature space.

We found that the key to successfully predicting the *E*_red._ and *E*^0^ values is the LUMO of products participating in the oxidation reactions (see Fig. S5†). Prediction of p*K*_a_ is strongly dependent on HOMOs. Overall, as shown in Fig. S4,† the reactant's HOMO and the product's LUMO participating in the oxidation reactions are inversely correlated to *E*_red._ and *E*^0^. Based on Koopmans' theorem, the ionization energy of molecules inversely correlates with their negative HOMO energies.^82^ There have been reports of a similar trend in recent years.^83,84^

### Structure-based descriptor

To create structure-based feature space (Descriptor FP), we (i) generate FPs from SMILES, and (ii) concatenate bit-strings from step (i) with the net charge of each sample. Before feeding the ML model, it is also critical to double-check for duplicates. In order to determine whether the bit-vector is long enough, we gather only the SMILES of the state QH_2_ and then check for duplicates *versus* bit length. A vector length of 1024 is needed to generate a distinct vector for each sample. Adding the electron charge states (0, ±1, ±2) will give us a feature space of 1025 dimensions for each sample.

**Fig. 4:**
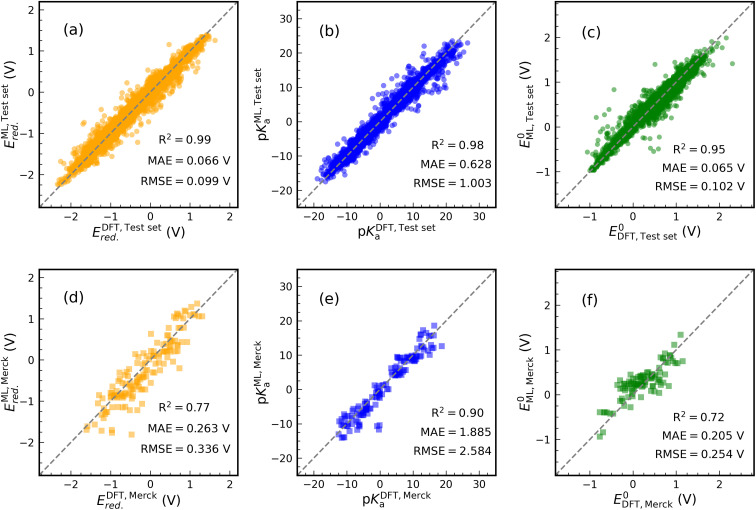
Scatter plots of the actual (DFT) values *versus* predicted values (ML) of *E*_red._, p*K*_a_, and *E*^0^. The ML models are trained in the structure-based feature space. The performance of the trained model is tested by internal ((a)–(c)) and external ((d)–(f)) data sets. In each case, the performance metrics were written inside the graph. *R*^2^ indicates the test set coefficient of determination. *E*_red._ and *E*^0^ were referenced to the SHE.

Fig. 4 shows the results of our predictive models when compared against the actual data. Each graph contains the performance metrics values (they are also in Table 1 with FP Descriptor). We use two data sets to validate the ML models: (i) an internal test set (20% of data set in each case) resulting from the splitting of the data set and (ii) an external data set containing 24 synthesized molecule structures purchasable from the Merck company website. The molecules of the external data set were sketched in Fig. S6.† DFT calculations are used to determine the thermodynamics of 144 samples (24 × 6 reactions) undergoing ET and PT reactions for the external data set. For PET, 96 samples (24 × 4 reactions) are evaluated. Only C=O subfragments of these molecules undergo protonation reactions.

The prediction of *E*_red._ achieved a *R*^2^ value of 0.99 on the internal test set (see Fig. 4(a)). Furthermore, MAE and RMSE reached values of 0.066 and 0.099 V, respectively, which are close to the performance of model 1 in Table 1. Training an ML model on ECFPs feature space produces a slightly better prediction for p*K*_a_ than model 4 (see Fig. 4(b)). There are values of 1.003, 0.628, and 0.98 for RMSE, MAE, and *R*^2^. Similar to other models, the structural model used to predict *E*^0^ delivers low RMSE and MAE values of 0.102 and 0.065 eV, respectively, accompanied by *R*^2^ = 0.95 (see Fig. 4(c)).

The performance of the structural models appeared to be good on the external test set (see Fig. 4(d)–(f)). In this case, for *E*_red._, *R*^2^, MAE, and RMSE are equivalent to 0.77, 0.263 V, and 0.336 V. For p*K*_a_ and *E*^0^ prediction, models result in *R*^2^ = 0.90, MAE = 1.885 V, RMSE = 2.584 V and *R*^2^ = 0.72, MAE = 0.205 V, RMSE = 0.254 V, respectively. The addition of some Merck compounds to our CompBatPET database would improve the model's predictability, although it requires extensive additional DFT calculations.

However, there are a few points to keep in mind regarding the prediction of the external test set: (i) the property-based feature space needs DFT-level computations to provide each attribute, but the structure-based descriptor only requires the SMILES of the molecule. Besides the significant difference in the computational cost, *i.e.* the former takes hours of CPU time while the latter requires only minutes, the SMILES are easily accessible in several chemistry software but DFT computations require more expertise. (ii) The simple backbones used to generate the database for training ML models can be thought of as the building blocks for more complicated ones, such as the Merck data set. (iii) The ML model prediction is still reliable enough to broadly screen the thermodynamics of PET reactions.

### Applicability of square-scheme and ML models

As the second test, we compute the Pourbaix diagram for 2,2-propionate ether anthraquinone (abbreviated 2,2PEAQ), which is recently experimentally investigated by Amini *et al.*^85^ Our database contains the core structure, but not the functional group in ref. 85. We only look at two-proton two-electron transfer reactions. Fig. S7† shows its molecular structure as well as the square-scheme representation of reactions. In addition to DFT, structural models using FPs were used to calculate the related quantities.

We consider 2,2PEAQ at the pH range of 0 to 14. In this range, direct protonation of 2,2PEAQ does not occur (path PT58 is closed). In the presence of the electrode, the first ET appears at a potential of −0.464 V_SHE_. Depending on the pH of the solution, ET or PT may occur in the following step. If pH ≤ 5, the reduction reaction is followed by a PT reaction for an applied potential between −0.464 and −0.762 V_SHE_. In contrast, a PET reaction (2,2PEAQ to 2,2PEAQ-H) is even more likely to occur under strongly acidic conditions, as indicated by the *E*^0^ value of −0.159 V_SHE_. According to the Nernst equation,^86^ which will be covered in more detail in the text that follows, this value drops by −0.059 V_SHE_ per pH at room temperature which makes a step-wise reaction (ET/PT) more likely around pH = 5. It is, then, thermodynamically favorable to convert 2,2PEAQ-H to 2,2PEAQ-H_2_ through a PET reaction. When 2,2PEAQ-H_2_ is in a reverse reaction towards 2,2PEAQ, two PET reactions are more likely to occur around pH = 5: despite the second PET always being favorable, the first PET (*e*.*g*. occurring at −0.352 V_SHE_ at pH = 0) becomes more favorable with an increment of +0.059 V per pH.

At 5 < pH < 8, the reduction reaction still takes place by incorporating 2 electrons and 2 protons along the ET-PET-PT path. The maximum applied potential required for the PET reduction reaction is −0.52 V_SHE_ occurring at pH of 8 (*i*.*e*., the reduction potential at pH = 0, −0.048 V, lowers by −0.059/pH). To oxidize 2,2PEAQ-H_2_ to 2,2PEAQ, the same 2PET reaction is anticipated to occur in the reversible pathway.

There are two electrons and one proton engaged through the ET-PET route for the reduction reaction at a pH range of 8 to 13. Eventually, there are only two electron steps in the very basic medium (pH > 13) and when applied potential is less than −0.763 V_SHE_.

The reduction potential *E*^0^ under nonstandard conditions depends on the activity of the reduced and oxidized species, which may differ from unity. The Nernst equation describes this deviation from the standard one as pH dependence8
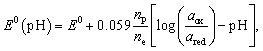
where *a*_ox_ and *a*_red_ indicate the activity of oxidized and reduced compounds, respectively. Additionally, *n*_p_ and *n*_e_ are the numbers of transferred protons and electrons in a reaction. Indeed, *E*^0^ indicates reduction potential at pH = 0. For this example, it is computed through

9 *E*^0^ = *E*^0^_PET04_ + *E*^0^_PET48_.

**Fig. 5:**
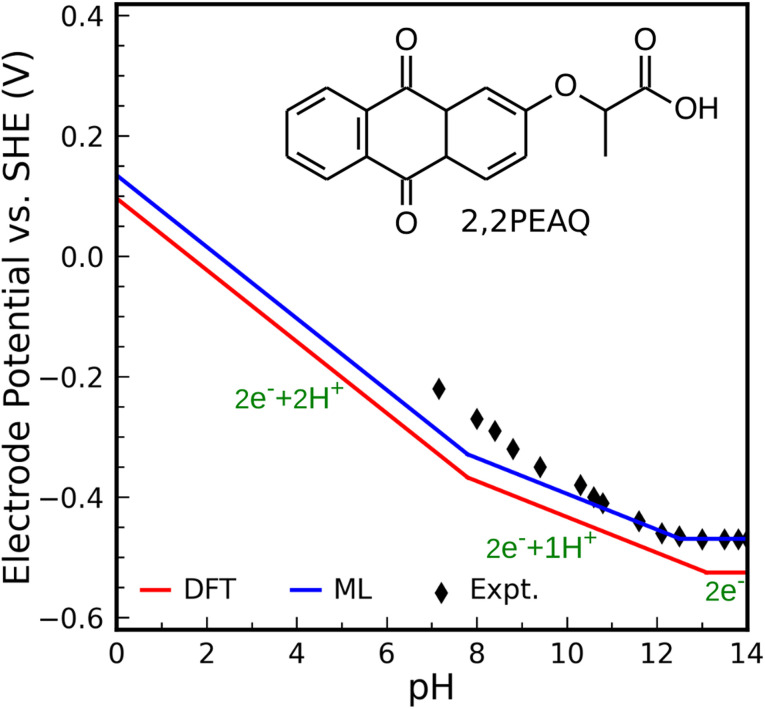
Predicted Pourbaix diagram of 2,2-propionate ether anthraquinone (2,2PEAQ) by DFT (red solid line) and ML models (blue solid line) *versus* experimental data.^85^

Fig. 5 shows the Pourbaix diagram of the 2,2PEAQ compound. When compared to the experimental data, both DFT and ML have excellent predictability. Small discrepancy relates to p*K*_a_ differences between different schemes. Despite calculations showing that two-proton two-electron dominates up to a p*K*_a_ of 8, the experiment suggests a similar reaction up to a p*K*_a_ of 10. A variation of this magnitude coincides with computational and predictive accuracy.

## Conclusion

To predict ET and PT processes a combined DFT-ML technique was used. We looked at a wide range of quinone type compounds at different charge and protonation states. For all these systems the redox potential and acidity constant were computed. We presented a dataset consisting of about 8200 compounds made up of 15 backbones decorated with 1–2 functional groups taken from a list of 9 groups. The data were extensively examined from a chemical and statistical perspective. As a result, we were able to train random forest models according to the structures and attributes. The molecular space can be described by chemical properties and/or structural characteristics. The most crucial features for the predictions of the acidity constant and redox potential, respectively, are the HOMO and LUMO energy levels. Strong predictability is demonstrated on the external test sets by models created using SMILES strings. While on the internal test sets, great accuracy was reached by all trained models. Although we tested the method on quinone derivatives, it applies to other types of redox compounds as well.

## Data availability

The Zenodo database contains the data for this paper, which can be downloaded at https://doi.org/10.5281/zenodo.7952777.

## Author contributions

Arsalan Hashemi: conceptualization, data curation, formal analysis, investigation, methodology, software, validation, writing – original draft. Reza Khakpour: methodology, software, review & editing. Amir Mahdian: writing – review & editing. Michael Busch: methodology, review & editing. Pekka Peljo: writing – review & editing. Kari Laasonen: conceptualization, resources, funding acquisition, project administration, supervision, writing – review & editing.

## Conflicts of interest

There are no conflicts to declare.

## Supplementary Material

DD-002-D3DD00091E-s001
