# Supplementary material for: Density functional theory and machine learning for electrochemical square-scheme prediction: an application to quinone-type molecules relevant to redox flow batteries
Source: Digit Discov. 2023 Sep 12;2(5):1565–76. doi: 10.1039/d3dd00091e (PMC10561546; doi:10.1039/d3dd00091e)
Supplement: DD-002-D3DD00091E-s001 [file DD-002-D3DD00091E-s001.pdf]

Electronic Supplementary Material (ESI) for Digital Discovery.
This journal is © The Royal Society of Chemistry 2023

Density Functional Theory and Machine Learning for Electrochemical Square-Scheme Prediction: An Application to Quinone-type Molecules Relevant to Redox Flow Batteries

Arsalan Hashemi,[*,†] Reza Khakpour,[†] Amir Mahdian,[†] Michael Busch,[‡] Pekka Peljo,[¶] and Kari Laasonen[†]

†*Department of chemistry and material science, School of chemical engineering, Aalto University, 02150 Espoo, Finland*

‡*Institute of theoretical chemistry, Ulm University, Albert-Einstein Allee 11, 89069 Ulm, Germany*

¶*Research Group of Battery Materials and Technologies, Department of Mechanical and Materials Engineering, Faculty of Technology, University of Turku, 20014 Turun Yliopisto, Finland*

E-mail: arsalan.hashemi@aalto.fi

Contents

1 Name of compounds presented in Figure 2 S3

2 Data distribution S4

3 Performance metrics S5

4 Spin-up orbitals vs. spin-down orbitals S6

5 Importance of MOs of reactants in training models S7

6 Correlation between key MOs and target variables S8

7 Importance of MOs of products in training models S9

8 Merck extracted strcutures S10

9 Example: 2,2-propionate ether anthraquinone (2,2PEAQ) S11

10 Python packages and dependencies S12

1 Name of compounds presented in Figure 2

Figure S1 shows the list of compounds used for the CompBatPET database construction. The commercialized name of these compounds is: *Compound 1*: Phthalic anhydride, *Compound 2*: [Bi-2,5-cyclohexadien-1-ylidene]-4,4'-Dione, *Compound 3*: 1,2-Benzoquinone , *Compound 4*: 1,4-Benzoquinone , *Compound 5*: 1,4-Naphthoquinone, *Compound 6*: Benzofuran-4,7-Dione, *Compound 7*: 2-Cyclopentene-1,4-Dione, *Compound 8*: Maleic anhydride, *Compound 9*: 2H-Pyran-2,5(6H)-Dione, *Compound 10*: 1,3-Dioxoindan, *Compound 11*: 9,10-Phenanthraquinone, *Compound 12*: 1,8-Pyrenedione, *Compound 13*: Acenaphthenequinone, *Compound 14*: Anthraquinone, *Compound 15*: 6,12-Chrysenedione.

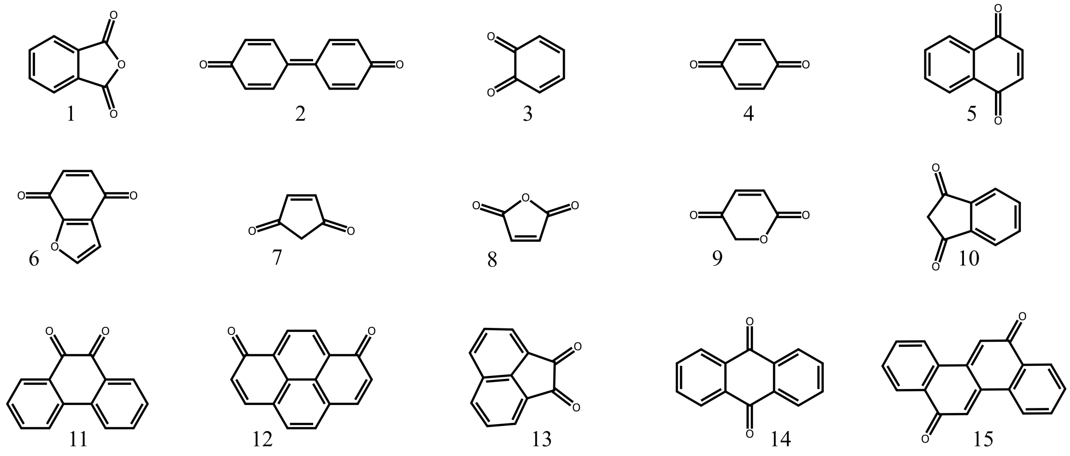

Figure S1: List of compounds accompanied by the 2D chemical structure depiction. Compounds are numbered from 1 to 15.

2 Data distribution

Table S1: Detailed descriptive statistics: mean, standard deviation (μ), minimum value (x_{min}), maximum value (x_{max}), lower quartile (Q_1), median, higher quartile (Q_3). There are 8214 samples (compounds) in the dataset that underwent different reactions.

Target variable	mean	μ	x_{min}	x_{max}	Q_1	median	Q_3
pK_a03	8.059	2.401	−2.721	22.396	7.038	8.273	9.154
pK_a14	−1.306	1.879	−10.207	4.616	−2.546	−1.189	0.128
pK_a25	−8.883	2.966	−21.992	0.095	−11.014	−8.576	−6.452
pK_a36	11.880	3.299	−2.836	27.626	10.365	11.369	13.212
pK_a47	4.909	1.821	−6.707	15.660	4.106	5.028	5.885
pK_a58	−4.206	2.686	−17.446	6.412	−6.040	−4.278	−2.293
$E_{red.}$01	0.872	0.366	−0.502	1.954	0.744	0.897	1.037
$E_{red.}$12	1.204	0.344	−0.134	2.426	1.041	1.203	1.372
$E_{red.}$34	0.314	0.356	−0.909	1.402	0.156	0.355	0.509
$E_{red.}$45	0.752	0.347	−0.401	2.027	0.589	0.768	0.919
$E_{red.}$67	−0.295	0.425	−1.629	1.696	−0.557	−0.246	−0.054
$E_{red.}$78	0.031	0.426	−1.312	2.180	−0.163	0.075	0.229
$E^0$04	0.794	0.385	−0.586	1.771	0.658	0.863	0.987
$E^0$15	0.675	0.365	−0.577	1.917	0.487	0.729	0.871
$E^0$37	0.412	0.409	−1.060	2.301	0.255	0.454	0.612
$E^0$48	0.323	0.458	−1.220	2.722	0.109	0.372	0.516

Table S1 reports the summary statistics for our data. There are 8213 samples (compounds) in the dataset that underwent different 6 ET, 6PT, and 4 PET reactions. $E_{red.}$, pK_a , and E^0 are the calculated target variables. Mean, standard deviation (μ), minimum value (x_{min}), maximum value (x_{max}), lower quartile (Q_1), median, higher quartile (Q_3) provide detailed information on the distribution of data.

3 Performance metrics

In this study, accuracy and performance refer to one or more metrics defined below. The formulas below denote N as the number of data points, $\hat{y}_i$ as the predicted value of ith sample, and y_i as the corresponding DFT (true) value:

- Coefficient of determination (R^2):

$$R^2 = 1 - \frac{\sum_{i=1}^{N}(y_i - \hat{y}_i)^2}{\sum_{i=1}^{N}(y_i - \bar{y}_i)^2},$$

  where

$$\bar{y} = \frac{1}{N}\sum_{i=1}^{N} y_i.$$

- Root Mean Squared Error (RMSE):

$$\text{RMSE} = \frac{1}{N}\sum_{i=1}^{N}(y_i - \hat{y}_i)^2.$$

- Mean Absolute Error (MAE):

$$\text{MAE} = \frac{1}{N}\sum_{i=1}^{N} |y_i - \hat{y}_i|.$$

4 Spin-up orbitals vs. spin-down orbitals

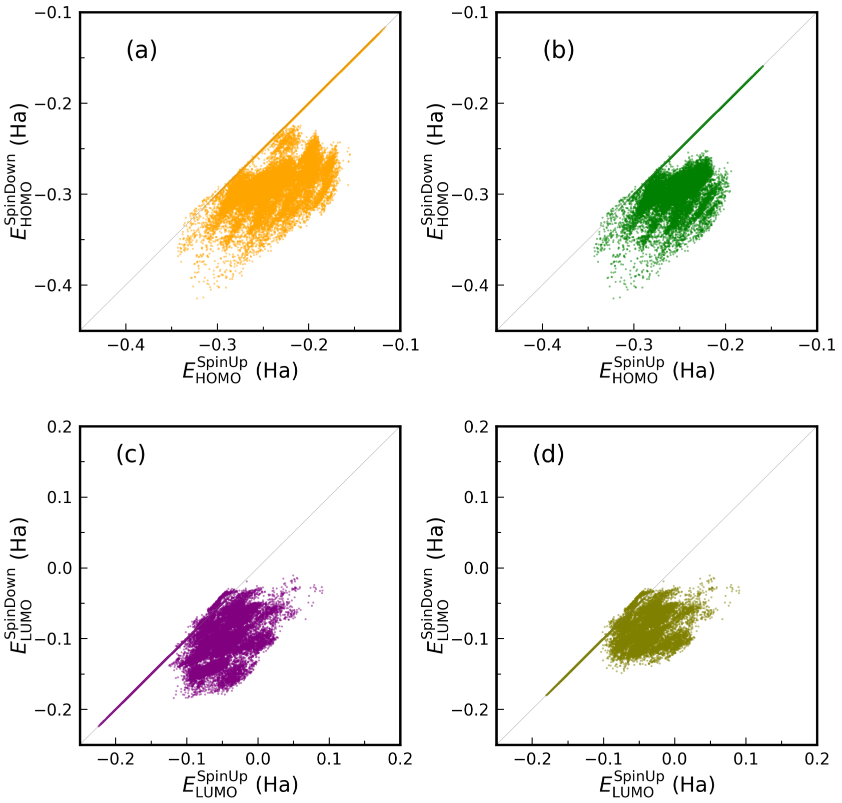

Figure S2: Comparing the energy of the spin-up and spin-down channels for (a) HOMOs of reactants participating in oxidation (ET) reactions, (b) HOMOs of reactants participating in PET reactions, (c) LUMOs of products produced by oxidation (ET) reactions, and (d) LUMOs of products produced by PET reactions.

When comparing the energy of the orbitals of the spin-up and spin-down channels, shown in Figure S2 (a) and (b), it can be shown that the HOMO spin-up channel has a greater energy level than the spin-down channel for the reactants involved in the ET and PET reactions. Therefore, when we refer to HOMO, we mean those spin-up channel orbitals. Figure S2 (c) and (d) illustrate similar plots for LUMOs of products from ET and PET reactions, demonstrating that orbitals in the spin-down channel serve as LUMO.

5 Importance of MOs of reactants in training models

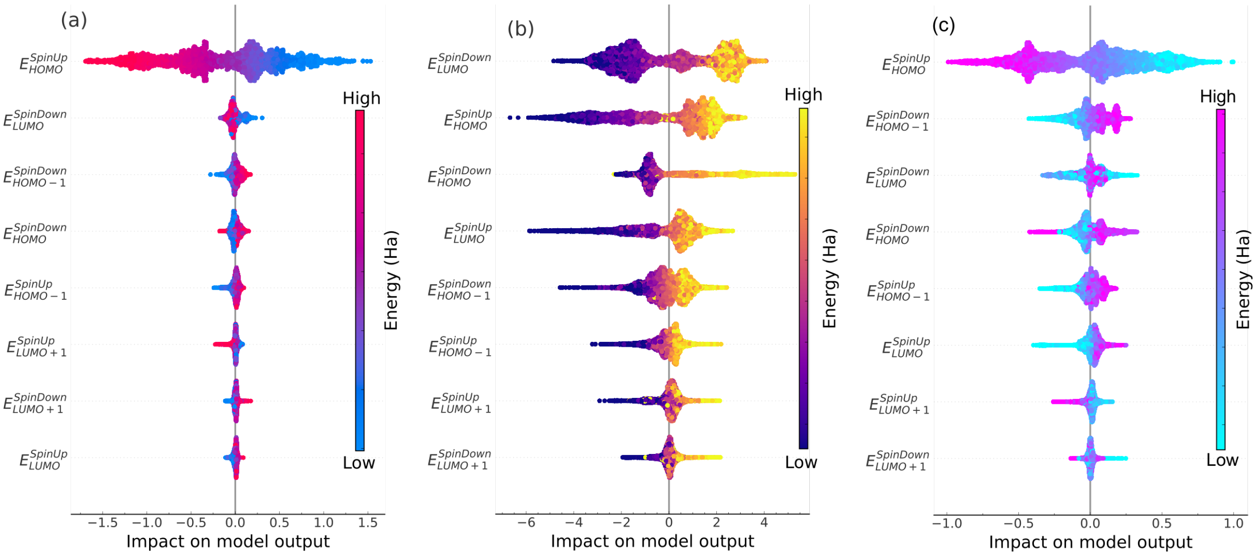

Figure S3: SHAP summary plot for elucidating the global feature influences on the (a) $E_{red.}$, (b) pK_a , and (c) E^0 trained models. Feature $\mathrm{E}_{\mathrm{y}}^{\mathrm{x}}$ indicates the energy of x orbital in y spin state. The baseline, positioned at zero, equals to an average target value in each case. The SHAP value (impact on model output) indicates the impact of feature missingness on the model prediction. The importance of the feature increases from bottom to top, e.g., $\mathrm{E}_{\mathrm{HOMO}}^{\mathrm{SpinUp}}$ and $\mathrm{E}_{\mathrm{LuMO}}^{\mathrm{SpinUp}}$ have the most and less importance in $E_{red.}$ prediction. In each plot, thousands of individual points from the training dataset are plotted, with a higher value being more pink/yellow/red, and a lower value is more cyan/purple/blue. This is depicted by the feature value bar on the right of each plot.

The importance of various feature variables extracted from reactants in training models can be seen in Figure S3. A SHAP value (impact on model output) 0 for a feature corresponds to the average prediction using all the other possible combinations of features except for the feature of interest. For instance, the SHAP value 0 for $\mathrm{E}_{\mathrm{HOMO}}^{\mathrm{SpinUp}}$ corresponds to the average prediction of models having different combinations of features (excluding $\mathrm{E}_{\mathrm{HOMO}}^{\mathrm{SpinUp}}$). SHAP value of 1 for a feature in Figure S3(a) means that the value of that feature increases the model's output by 1. Our results show that $\mathrm{E}_{\mathrm{HOMO}}^{\mathrm{SpinUp}}$ is the most important feature for training the models that predicted $E_{red.}$ and E^0. Indeed, those orbitals placed at the edge of the HOMO-LUMO gap play a crucial role in the feature space used for pK_a prediction.

6 Correlation between key MOs and target variables

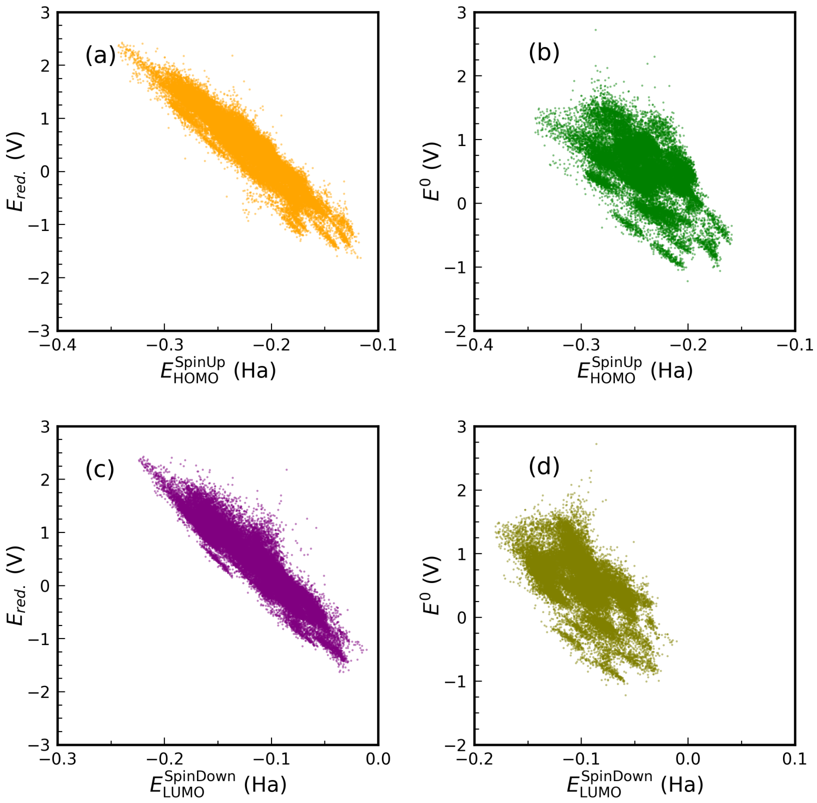

Figure S4: Relationship between key features evaluated by SHAP and the target variables: (a) E_{HOMO}^{SpinUp} vs. $E_{red.}$, (b) E_{HOMO}^{SpinUp} vs. E^0, (c) $E_{LUMO}^{SpinDown}$ vs. $E_{red.}$, and (d) $E_{LUMO}^{SpinDown}$ vs. E^0. The reactants involved in the ET and PET reactions, respectively, were used to extract the features used in (a) and (b). While the features employed in (c) and (d) were extracted from the products resulting from the ET and PET processes, respectively.

Figure S4 shows the correlation between the most crucial features (MOs) for predicting $E_{red.}$ and E^0. The reactant's HOMO and the product's LUMO participating in the oxidation reactions are inversely correlated to $E_{red.}$ and E^0.

7 Importance of MOs of products in training models

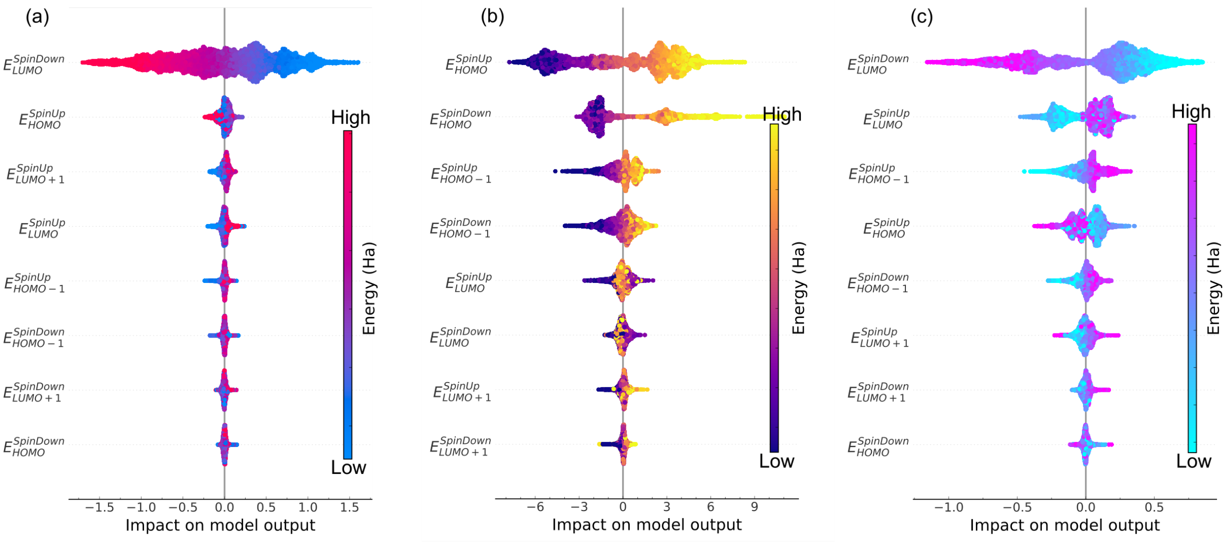

Figure S5: SHAP summary plot for elucidating the global feature influences on the (a) $E_{red.}$, (b) pK_{a} , and (c) E^0 trained models. Feature $\mathrm{E}_{\mathrm{y}}^{\mathrm{x}}$ indicates the energy of x orbital in y spin state. The baseline, positioned at zero, equals an average target value in each case. The SHAP value (impact on model output) indicates the impact of feature missingness on the model prediction. The importance of the feature increases from bottom to top. In each plot, thousands of individual points from the training dataset are plotted, with a higher value being more pink/yellow/red, and a lower value being more cyan/purple/blue. This is depicted by the feature value bar on the right of each plot.

Figure S5 illustrates how the target variables are related to the features of products. By taking into account a product's attributes, $E_{red.}$ and E^0 are inversely related to $\mathrm{E}_{\mathrm{LUMO}}^{\mathrm{SpinDown}}$. Additionally, the p$K_{\mathrm{a}}$ prediction is highly sensitive to HOMOs.

8 Merck extracted strcutures

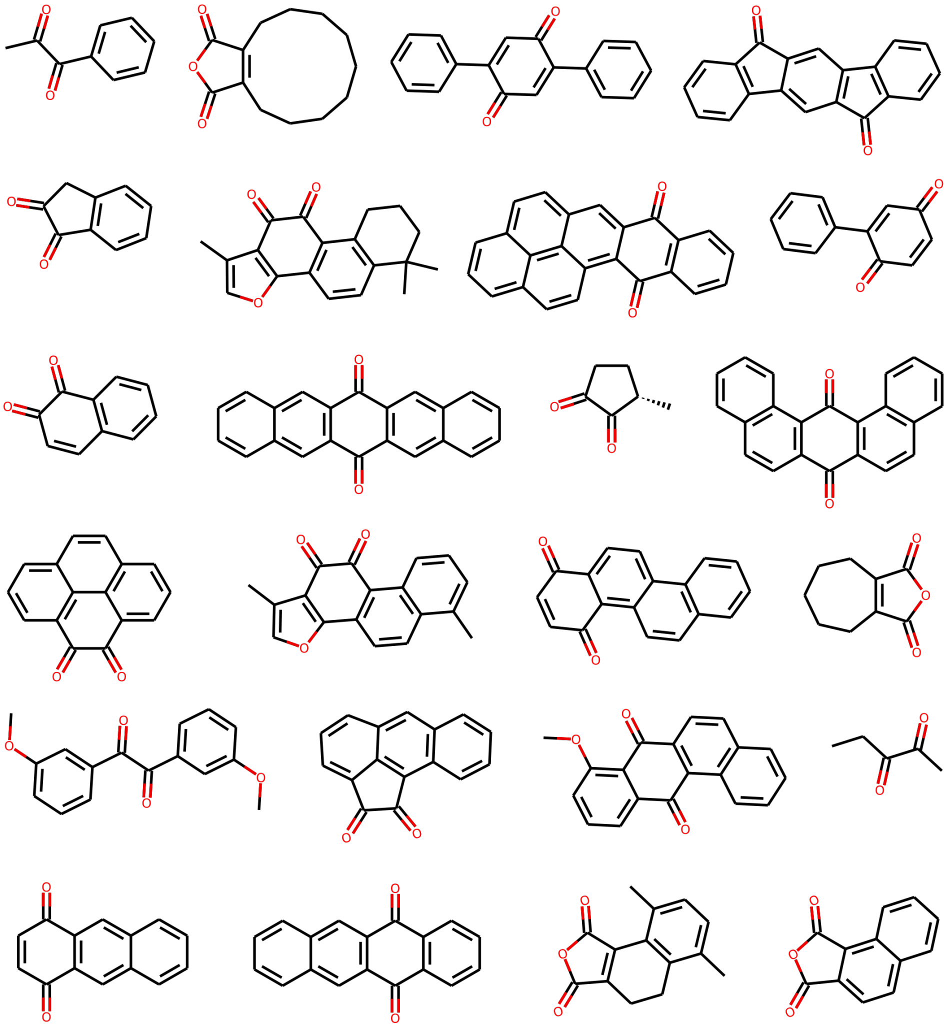

Figure S6: Experimentally synthesized molecules extracted from Merck.

A list of the compounds utilized to generate the external data set for ML model validations is shown in Figure S6. We selected these compounds based on their (i) ability to go through similar reduction reaction processes, i.e., their ketone group content, and (ii) physical availability. To arrive at this, we browsed Merck's website.

9 Example: 2,2-propionate ether anthraquinone (2,2PEAQ)

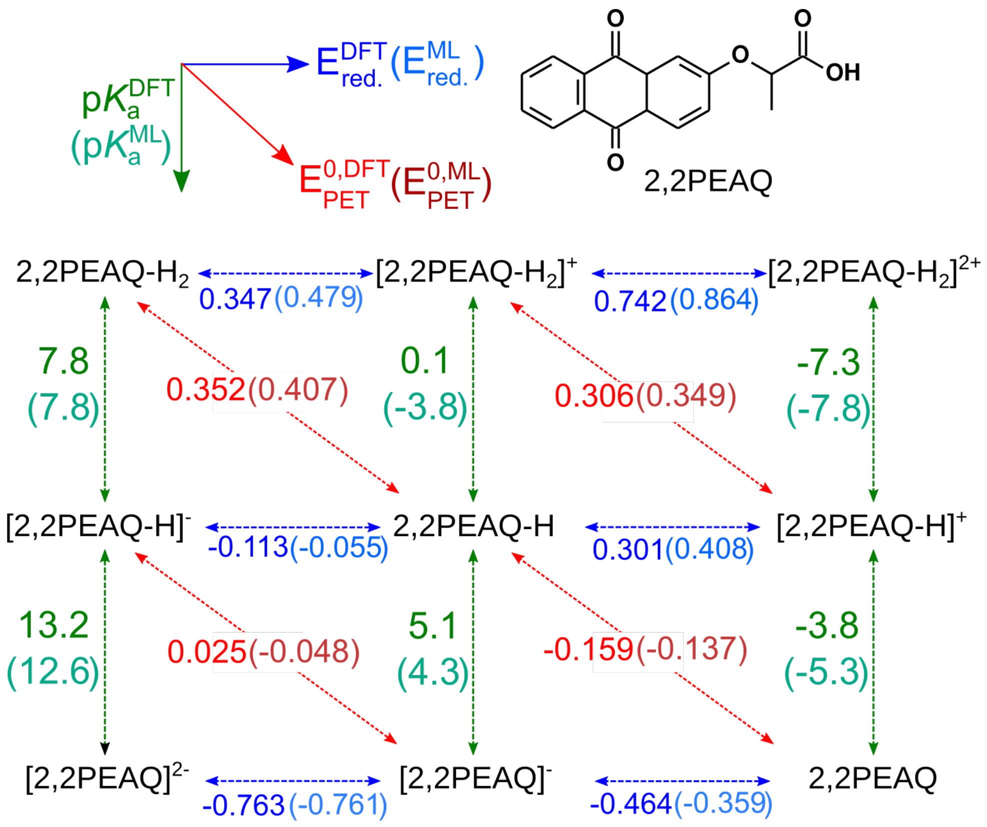

Figure S7: Square representation for 2,2PEAQ-H_2 oxidation reactions. On top, you see the structure of 2,2PEAQ. The horizontal direction indicates an ET reaction. Numbers are oxidation potential in V. The vertical direction indicates PT (acid/base reaction constant). Numbers are pK_a which are unitless. The diagonal direction indicates proton-coupled electron transfer reduction potential (V). ML models were used to predict the numbers in parentheses, where ECFPs are descriptors.

Figure S7 shows the schem of squares representations of 2,2PEAQ species undergoing the reaction below:

$$2,2\text{PEAQ} - \text{H}_2 \rightleftharpoons 2,2\text{PEAQ} + 2\,\text{H}^+ + 2\,\text{e}^- . \quad \text{(S1)}$$

2,2PEAQ is an abbreviation for 2,2-propionate ether anthraquinone and $2,2\text{PEAQ} - \text{H}_2$ represents the fully reduced form of the compound.

10 Python packages and dependencies

To successfully run the Jupyter notebook, Python 3.10 or higher is required. Previous versions might also work fine. We leave it to the readers to check. Indeed, the following libraries are required to be installed. The dependencies found in PyPI.

shap==0.42.1

numpy==1.24.3

pandas==2.0.2

matplotlib==3.7.1

natsort==8.4.0

pathlib==1.0.1

scikit-learn==1.2.2

jupyterlab==4.0.1

ase==3.22.1

seaborn==0.12.2

rdkit==2023.3.1

scipy==1.10.1

In addition "README" and "requirements.txt" files are included in the folder "03_pynb_scripts" that was submitted to the Zenodo DB, to instruct for an easy installation of the packages.
